# Fibroblast Growth Factor-7 and Hair Biology: Bridging Basic Science and Therapeutic Applications

**DOI:** 10.3390/cimb48010102

**Published:** 2026-01-19

**Authors:** Huey-Chun Huang, Wang-Ju Hsieh, Ivona Percec, Tsong-Min Chang

**Affiliations:** 1 Department of Medical Laboratory Science and Biotechnology, China Medical University, Taichung City 406040, Taiwan; 2 Schweitzer Biotech Company, Taipei City 114066, Taiwan; 3 Division of Plastic Surgery, Department of Surgery, University of Pennsylvania, Philadelphia, PA 19010, USA; 4 Department of Applied Cosmetology, Hungkuang University, Taichung City 433304, Taiwan

**Keywords:** hair growth, FGF-7, KGF, growth factor, hair follicle, alopecia, hair loss, exosome-based drug delivery, molecular pathways

## Abstract

Alopecia profoundly impacts psychological well-being and quality of life, yet current therapeutic options such as minoxidil and finasteride exhibit limited efficacy. Fibroblast growth factor 7 (FGF-7), also known as keratinocyte growth factor (KGF), is a paracrine growth factor secreted by dermal papilla cells that specifically activates the epithelial receptor FGFR2b. Receptor engagement triggers multiple downstream signaling cascades, including the MAPK/ERK, PI3K/Akt, and Wnt/β-catenin pathways, promoting keratinocyte proliferation, stem cell activation, and the transition of hair follicles into the anagen phase. Both in vitro and in vivo animal studies consistently demonstrate that FGF-7 accelerates telogen-to-anagen transition and enhances follicular regeneration. FGF-7 acts synergistically with insulin-like growth factor 1 (IGF-1) and vascular endothelial growth factor (VEGF) to sustain nutrient delivery and cell proliferation. Human scalp studies further reveal a strong association between the FGF-7/FGFR2b signaling and follicular activity; however, clinical trials remain scarce. Topical application of FGF-7 has demonstrated an excellent safety profile, whereas systemic administration necessitates careful monitoring. Future directions include the development of engineering to extend the systemic half-life, advanced delivery systems, and gene or mRNA-based therapeutic approaches. Thus, the FGF-7/FGFR2b axis is a highly compelling molecular target for next-generation hair regeneration therapies.

## 1. Introduction

Alopecia extends beyond a cosmetic concern; it profoundly affects psychological well-being and overall quality of life. The most common types include androgenetic alopecia (AGA) [1], telogen effluvium (TE) [2] and chemotherapy-induced alopecia (CIA) [3]. Among these, AGA represents the most prevalent form of non-scarring hair loss in both men and women. The resulting hair thinning and altered appearance frequently lead to diminished self-esteem, heightened depression and anxiety, and, in severe cases, social withdrawal. Systematic reviews and meta-analyses further demonstrate that individuals with AGA typically exhibit moderate impairment in health-related quality of life, with self-esteem and emotional well-being being disproportionately affected [4].

In the case of CIA, approximately 65% of cancer patients experience hair loss, typically beginning one to two weeks after treatment initiation and worsening with subsequent cycles [5,6]. CIA is widely recognized as one of the most distressing adverse effects of cancer therapy, as it alters appearance and body image, leading to emotional distress, social difficulties, and, in some cases, even reduced adherence to treatment [7]. In a cohort study of breast cancer patients, 97.2% reported that hair loss had a substantial impact on their quality of life, compromising emotional well-being, daily functioning, and social interactions [8]. Currently, the U.S. Food and Drug Administration (FDA) has approved only two pharmacological treatments for AGA: minoxidil and finasteride. Minoxidil, a topical vasodilator, prolongs the anagen (growth) phase of hair follicles but requires continuous, long-term application, and its effects diminish rapidly upon discontinuation. Common adverse effects include scalp irritation, contact dermatitis, and hypertrichosis [9]. Finasteride, an oral 5α-reductase type II inhibitor, reduces dihydrotestosterone (DHT) levels, thereby attenuating follicular miniaturization and promoting increased hair density [10]. However, its safety profile remains controversial, particularly regarding sexual adverse effects such as decreased libido, erectile dysfunction, and reduced semen volume, some of which may persist following treatment cessation [10]. Consequently, the modest efficacy and potential side effects of current therapies restrict their widespread clinical applicability.

**Figure 1:**
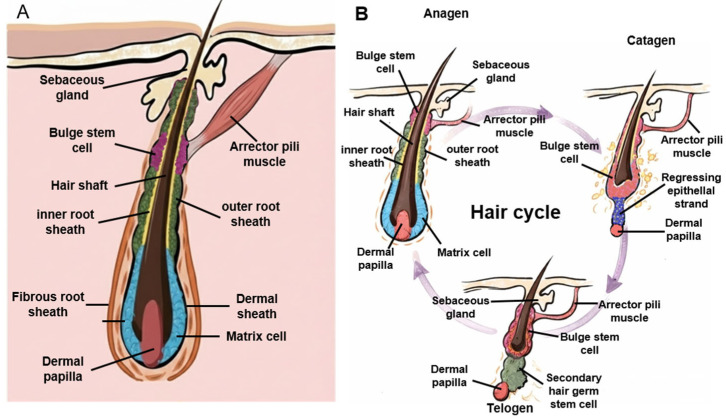
Hair follicle anatomy and hair growth cycle. (**A**) Schematic illustration of the human hair follicle during the anagen phase, highlighting the major anatomical compartments and key cellular components. The hair follicle is composed of the hair shaft, inner root sheath (IRS), outer root sheath (ORS), fibrous root sheath, and dermal sheath. The bulge region, located at the insertion site of the arrector pili muscle, harbors hair follicle stem cells (HFSCs). The dermal papilla (DP), a specialized mesenchymal structure at the base of the follicle, interacts with matrix cells to regulate hair shaft production and follicular growth. The sebaceous gland and arrector pili muscle are shown as integral components of the pilosebaceous unit. (**B**) Diagrammatic representation of the hair growth cycle, illustrating the dynamic morphological changes in the hair follicle during anagen (growth phase), catagen (regression phase), and telogen (resting phase). During anagen, the follicle elongates and the dermal papilla is positioned deep within the bulb, supporting active matrix cell proliferation. In catagen, epithelial regression occurs, forming a regressing epithelial strand and repositioning the dermal papilla toward the bulge region. In telogen, the follicle enters a quiescent state, with the dermal papilla closely associated with the bulge and secondary hair germ, enabling subsequent re-entry into anagen.

Importantly, these alopecia subtypes differ in their effects on follicular epithelial compartments and hair follicle stem cell (HFSC) dynamics, and most do not imply that the hair follicle is biologically “dead.” In Figure 1, the hair follicle anatomy and hair growth cycle are illustrated [11]. AGA is a non-scarring miniaturization disorder in which bulge HFSC populations are largely preserved, whereas the progenitor cell pool and epithelial output are progressively reduced, consistent with impaired HFSC-to-progenitor conversion rather than follicle loss [12]. TE likewise represents a non-scarring and typically reversible disturbance of the hair cycle, characterized by premature termination of anagen and synchronized entry into telogen, without destruction of follicular epithelial architecture [13]. In contrast, CIA primarily reflects acute cytotoxic injury to rapidly proliferating matrix keratinocytes during anagen; although hair regrowth commonly occurs after treatment cessation, persistent or permanent CIA has been associated with additional damage to the bulge epithelial stem cell compartment, which may compromise regenerative capacity [14,15].

The regulation of the hair follicle growth cycle is governed by a complex interplay of multiple growth factors, including insulin-like growth factor-1 (IGF-1), vascular endothelial growth factor (VEGF), epidermal growth factor (EGF), and fibroblast growth factor (FGF) [16,17,18,19]. In particular, FGF-7 is predominantly secreted by dermal papilla cells (DPCs) and functions in a paracrine manner to regulate the hair follicle epithelium by binding with high specificity to FGFR2b expressed on keratinocytes and follicular epithelial cells [20,21]. Through this ligand–receptor interaction, FGF-7 signaling directly promotes keratinocyte proliferation and differentiation while maintaining the structural and functional integrity of the follicular epithelium during hair follicle regeneration. This review provides a comprehensive overview of the latest advances in FGF-7 research on hair growth and explores its potential therapeutic and cosmetic applications, aiming to establish a solid scientific foundation for future research and translational development [20,22].

## 2. Biology of FGF-7

### 2.1. Structure and Expression

FGFs are classified according to their mode of action into paracrine, endocrine, and intracrine types. FGF-7, a paracrine member of the FGF family, belongs to the FGF-7 subfamily along with FGF-10 and FGF-22, characterized by its selective activation of the FGFR2b isoform [23]. Genetically, the human FGF-7 gene is located on chromosome 15q15–21.1 and encodes a secreted protein containing an N-terminal signal peptide. Structurally, FGF-7 adopts a β-trefoil fold stabilized by multiple disulfide bonds, a characteristic feature of FGF protein family [24]. Recent evidence by Niu et al. demonstrated that DPCs secrete FGF-7, which regulates the proliferation and differentiation of both DPCs and hair follicle stem cells (HFSCs) via a paracrine mechanism [25]. Similarly, Kinoshita-Ise et al. reported broad FGF-7 expression in human scalp-derived fibroblasts, DPCs, and sheath cells, further reinforcing its mesenchymal origin [26]. Although keratinocytes themselves produce little FGF-7, they express FGFR2b and thus serve as the principal targets of FGF-7-mediated signaling [27].

Using RNA in situ hybridization, Rosenquist & Martin demonstrated that FGF-7 is strongly expressed in the dermal papilla during the anagen phase of mouse hair follicles, declines markedly by late anagen VI, and becomes nearly undetectable during catagen (regression) and telogen (resting) phases [20]. Consistent with this pattern, Kawano et al. showed that FGF-7 expression peaks during anagen and progressively decreases throughout catagen and telogen phases [28].

### 2.2. Receptors and Signaling Pathways

**Figure 2:**
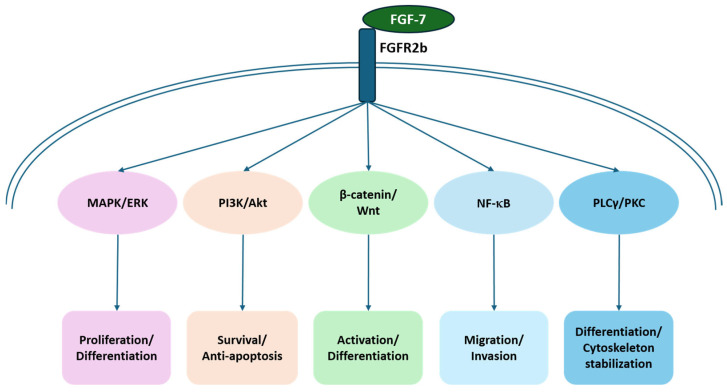
Schematic representation of FGF-7/FGFR2b signaling pathways and their downstream cellular effects. Upon FGF-7 binding to its cognate FGFR2b, several intracellular signaling cascades are activated, including the MAPK/ERK pathway (promoting proliferation and differentiation), the PI3K/Akt pathway (mediating cell survival and anti-apoptotic responses), the β-catenin/Wnt pathway (regulating cellular activation and differentiation), the NF-κB pathway (governing cell migration and invasion), and the PLCγ/PKC pathway (involved in differentiation and cytoskeleton stabilization). Together, these signaling axes orchestrate a coordinated network of cellular responses that collectively drive proliferation, survival, activation, migration, and cytoskeletal reorganization.

The biological effects of FGF-7 are primarily mediated through its cognate receptor, FGFR2b, an epithelial splice variant that is abundantly expressed in keratinocytes and hair follicle epithelial cells. FGFR2b is widely distributed in the skin and hair follicle epithelium, where it plays a critical role in regulating keratinocyte differentiation. Its ligand, FGF-7, is predominantly secreted by mesenchymal-derived cells [27,29,30]. Upon binding of FGF-7 to FGFR2b, multiple downstream signaling cascades are triggered, as illustrated in Figure 2:

1. MAPK/ERK cascade: Promotes the proliferation and differentiation of keratinocytes and hair follicle epithelial cells [31].
2. PI3K/Akt pathway: Enhances cell survival and confers resistance to apoptosis, thereby maintaining the structural integrity of the follicular epithelium [32].
3. β-catenin/Wnt pathway: FGF-7 is suggested to interact with the Wnt/β-catenin signaling pathway to modulate the activity of HFSCs. Studies have reported that FGF-7 secreted by DPCs can activate Wnt signaling, thereby promoting HFSCs activation and differentiation [25].
4. NF-κB pathway (context-dependent): FGF-7 has been reported to activate NF-κB signaling in non-cutaneous epithelial systems, leading to increased expression of VEGF and matrix-remodeling genes. However, direct evidence for FGF-7–induced NF-κB activation in human scalp or hair follicle models is currently limited, and its relevance to hair follicle regeneration should therefore be regarded as hypothesis-generating pending validation in follicle-specific systems [33].
5. PLCγ/PKC pathway (epithelial differentiation context): FGFR2b signaling can engage PKC isoforms to regulate keratinocyte differentiation and epithelial homeostasis [27,34]. While this pathway is well supported in epithelial biology, direct evidence linking it to human hair shaft elongation or hair cycle control remains limited, suggesting an indirect role through maintenance of a supportive follicular microenvironment.

In the context of hair growth regulation, FGF-7 functions within a broader pro-regenerative growth-factor network that includes IGF-1 and VEGF. IGF-1 has been shown to directly stimulate human hair follicle growth in organ culture [35], whereas VEGF primarily supports follicle viability by enhancing perifollicular vascular supply [17]. At present, however, direct experimental evidence demonstrating true pharmacologic synergy between exogenous FGF-7 and IGF-1 or VEGF within the same hair follicle system remains limited. Accordingly, these interactions are more accurately described as functional complementarity and potential pathway convergence within the hair follicle regulatory network, pending further mechanistic validation. Collectively, the signaling network mediated by FGF-7 and its receptor FGFR2b not only directly promotes the proliferation and survival of follicular epithelial cells but also cooperates with IGF-1- and VEGF-dependent pathways to modulate the follicular microenvironment, thereby playing a pivotal role in hair follicle regeneration.

## 3. Role of FGF-7 in Hair Biology

### 3.1. Regulation of Hair Cycle

The hair growth cycle consists of three phases: anagen, catagen, and telogen, with transitions between these stages tightly regulated by a complex interplay of signaling factors [36]. FGF-7 is recognized as a key anagen-inducing growth factor that enhances signaling communication between DPCs and follicular epithelial or stem cells, thereby facilitating the transition of hair follicles into the active growth state [25]. Early studies demonstrated that exogenous FGF-7 stimulates the proliferation of follicular epithelial cells and promotes hair regeneration [37]. Subsequent investigations revealed that growth factors such as VEGF and FGF-7 may act in a complementary manner, contributing to anagen maintenance and hair cycle progression through distinct yet convergent biological processes, including epithelial proliferation and microenvironmental support [17]. Collectively, the ability of FGF-7 to facilitate telogen-to-anagen transition and support anagen progression highlights its relevance to hair follicle regeneration and its potential applicability in therapeutic strategies targeting hair growth disorders.

### 3.2. Effects on Hair Follicle Cells

#### 3.2.1. Keratinocytes

FGF-7 was originally identified as a potent stimulator of epidermal keratinocyte proliferation and differentiation, as well as an activator of follicular epithelial cell function. Early studies demonstrated that exogenous FGF-7 accelerates keratinocyte proliferation and induces the expression of differentiation markers, thereby promoting hair follicle regeneration and elongation of the hair shaft [27,37].

#### 3.2.2. Dermal Papilla Cells (DPCs)

Although FGF-7 is predominantly secreted by DPCs and acts on follicular epithelial cells via paracrine signaling, emerging evidence suggests that it can also exert autocrine effects within DPCs. Niu et al. demonstrated that DPC-derived FGF-7 not only helps preserve the stemness of DPCs but also promotes their proliferation and contributes to hair follicle regeneration through activation of the Wnt signaling pathway [25].

#### 3.2.3. Bulge Region Stem Cells

HFSCs reside primarily within the bulge region, serving as the principal reservoir for hair follicle regeneration. Accumulating evidence indicates that FGF-7 indirectly activates these stem cells through paracrine signaling between DPCs and follicular epithelial cells, thereby promoting their transition into a proliferative state and initiating a new hair follicle cycle. Furthermore, FGF-7/FGFR2b signaling has been shown to play a pivotal role in HFSC activation and in orchestrating hair follicle cycle regeneration [38,39].

#### 3.2.4. Crosstalk with Other Growth Factors in Hair Follicle Cycle

The hair follicle represents a classic model of mesenchymal–epithelial interactions, in which cyclical transitions through anagen, catagen, and telogen are tightly regulated by a complex network of multiple growth factors. These signaling molecules not only regulate cellular proliferation and differentiation but also collectively determine the follicle’s regenerative capacity, vascularization, and resilience to oxidative stress and cellular damage. Among them, the FGF family, together with several non-FGFs, including IGF-1, VEGF, EGF, PDGF, TGF-β, and IL-1, constitutes the core regulatory signaling network within the follicular microenvironment. The specific roles of FGF-7 and other growth factors in the hair growth cycle are summarized in Table 1.

IL-1, secreted by keratinocytes, can upregulate FGF-7 transcription in fibroblasts, forming a dual paracrine positive feedback loop (“IL-1 → FGF-7 → keratinocytes”) that facilitates follicle repair and regeneration following skin injury or inflammation [51]. In contrast, the inhibitory actions of FGF-5 and FGF-18 balance the activation: FGF-5 expression increases during late anagen to induce catagen [42,43], whereas FGF-18 is highly expressed during telogen, maintaining the resting phase and preventing premature entry into anagen [28]. This dynamic interplay between stimulatory and inhibitory cues ensures the proper temporal progression and homeostatic balance of the hair cycle. Additionally, FGF-9, secreted by γδ T cells during wound healing and skin remodeling, activates mesenchymal Wnt signaling and induces wound-induced hair neogenesis (WIHN) [44]. FGF-10 and FGF-22, although primarily acting on the follicular epithelium and the inner root sheath, contribute to follicle morphogenesis and outer root sheath organization during embryonic development and early regeneration [45,46]. Finally, FGF-1 and FGF-2 (aFGF/bFGF) exert supportive roles by promoting DPC proliferation and enhancing their inductive potential, synergizing with PDGF-AA to sustain dermal papilla cell functionality [41].

Non-FGF growth factors primarily function to sustain cellular energy metabolism and regulate the follicular microenvironment. IGF-1, predominantly secreted by DPCs, activates the PI3K/Akt and MAPK/ERK pathways via the IGF-1 receptor (IGF1R), thereby promoting the proliferation of follicular epithelial and matrix cells, suppressing apoptosis, and prolonging the anagen phase. IGF-1 also upregulates VEGF expression to enhance perifollicular microcirculation, delays the transition into catagen, and increases the Bcl-2/Bax ratio, improving follicular vascular support [16,35,47,52]. Although a direct synergistic mechanism between IGF-1 and FGF-7 in mediating dermal–epithelial signaling during hair shaft elongation has not been explicitly demonstrated, co-treatment with both factors in skin and epithelial models enhances keratinocyte survival and migration, suggesting potential cooperative effects [53]. Both IGF-1 and FGF-7 converge on downstream ERK and AKT signaling cascades, acting synergistically to promote epithelial regeneration and maintenance of the anagen phase within the follicular microenvironment [31,37]. In human hair follicle organ culture systems and animal or skin models, IGF-1 has been shown to maintain or induce anagen and stimulate linear hair growth [35,47,54], whereas FGF-7 acts as a critical endogenous factor driving follicular growth and differentiation in mouse and rodent models [37]. These findings indicate that IGF-1 and FGF-7 act synergistically to promote hair follicle growth and regeneration. VEGF is recognized as a key regulator of hair follicle activity during the anagen phase, primarily by promoting perifollicular angiogenesis to enhance oxygen and nutrient delivery. In a mouse model, Yano et al. demonstrated that overexpression of VEGF in outer root sheath keratinocytes markedly increased perifollicular vascularization and accelerated hair regeneration, whereas treatment with anti-VEGF antibodies delayed follicular growth and reduced follicle size [17]. Moreover, VEGF can act directly on DPCs, activating the ERK signaling pathway via VEGFR-2 to stimulate DPC proliferation (VEGF → DPC proliferation) [55]. Consistent with these findings, Mecklenburg et al. reported a significant increase in skin microvasculature upon the transition of follicles into anagen, demonstrating the close association between angiogenesis and follicular growth [56]. Taken together, these observations suggest that VEGF and FGF-7 exert complementary roles in the hair follicle microenvironment: VEGF primarily sustains nutrient supply and microvascular integrity, whereas FGF-7 promotes follicular epithelial cell proliferation. Together, they cooperatively facilitate the initiation and maintenance of the anagen phase in hair follicles.

EGF exerts a dual regulatory role in the hair follicle cycle. While it promotes keratinocyte proliferation and tissue repair, excessive EGF activity has been shown to inhibit anagen initiation and induce follicular regression or miniaturization [18,48]. Moderate EGF levels support epithelial renewal and regeneration; however, sustained or elevated exposure can trigger a catagen-like state. In CIA models, appropriate EGF pretreatment has been shown to confer protective effects against follicular damage [49]. In contrast, FGF-7 acts through FGFR2b on follicular epithelial cells and keratinocytes, stimulating proliferation and regeneration [37]. These findings suggest that EGF and FGF-7 may function in an antagonistic or competitive manner within follicular epithelial signaling, and maintaining a proper balance between these pathways is crucial for normal hair cycle dynamics [57]. Furthermore, platelet-derived growth factor (PDGF), produced by platelets and stromal cells, acts synergistically with FGF-2 to sustain dermal papilla inductive activity, thereby reinforcing extracellular matrix remodeling and structural integrity within the follicular mesenchyme [41].

In contrast, TGF-β1/β2 function as classic negative regulatory signals within the hair follicle cycle, inducing epithelial apoptosis and promoting the transition into catagen, effectively acting as a biological “brake” in the hair follicle cycle. Excessive TGF-β expression can precipitate premature follicular regression, whereas moderate inhibition has been shown to prolong the anagen phase [50]. Consequently, most hair growth–promoting strategies aim to enhance pro-growth mediators such as FGF-7, IGF-1, and VEGF, while concurrently attenuating the inhibitory TGF-β axis, which plays a pivotal role in follicular regeneration and anagen maintenance. Overall, the hair follicle microenvironment operates as a multichannel, integrated, oscillatory regulatory system rather than a linear cascade. Within this network, FGF-7, IGF-1, and VEGF constitute the core pro-growth axis, driving epithelial cell proliferation and angiogenic support; EGF and PDGF provide auxiliary functions in epithelial repair and mesenchymal remodeling; FGF-5, FGF-18, and TGF-β constitute the negative feedback loop, ensuring proper cycle termination and re-initiation; and IL-1 acts as a cross-cellular amplifier, reinforcing regenerative signaling via FGF-7 induction [40]. Together, these interconnected pathways enable hair follicles to maintain dynamic equilibrium in response to internal and external stimuli- such as injury, chemotherapy, or inflammation, thereby sustaining the defining features of hair follicle regeneration: self-limited proliferation and a reversible cyclical pattern.

## 4. Experimental and Clinical Evidence

### 4.1. Functional Roles of FGF-7 in Hair Follicle Organ Culture and Cellular Models

The ex vivo hair follicle organ culture model, first established by the Philpott group, represents a canonical three-dimensional mini-organ system that accurately recapitulates the dynamic phases of the hair growth cycle. This model has been widely utilized to assess the effects of exogenous factors on hair follicle growth and regeneration [58]. Subsequent studies using this model have demonstrated that FGF signaling serves as a key positive regulator of hair follicle elongation and cycle progression. In particular, FGF-7 expression is markedly elevated during the anagen phase, where it confers cytoprotective effects on follicular epithelial cells. Organ culture experiments further reveal that exogenous FGF-7 significantly enhances hair follicle elongation and supports follicles in a prolonged growth state, thereby providing compelling evidence for the stimulatory role of FGF-7 within the follicular microenvironment [37,59].

At the cellular level, FGF-7 has been shown to directly stimulate keratinocyte proliferation and migration. Early studies reported that recombinant human FGF-7 markedly enhances the random migration of normal keratinocytes and increases urokinase-type plasminogen activator activity in a dose-dependent manner. These effects are abrogated by neutralizing antibodies, confirming the specificity of FGF-7 action [60]. Further mechanistic studies revealed that FGF-7/FGFR2b signaling promotes epithelial cell migration through a pathway involving ADAM17 and EGFR/ERK1/2 activation [61].

Moreover, DPCs and fibroblasts have been identified as the principal sources of FGF-7. Acting in a paracrine manner, FGF-7 targets follicular epithelial and stem cells, establishing a characteristic mesenchymal–epithelial signaling network [62,63,64]. Recent co-culture studies have further demonstrated that FGF-7 secreted by DPCs not only activates follicular epithelial cells but also sustains DPC stemness and proliferation via an autocrine feedback mechanism, supporting its central role in hair follicle regeneration [25].

Quantitative comparisons among FGFs in human hair follicle organ culture remain limited. In human scalp hair follicle organ culture models, exogenous FGF-7 (KGF-1) has been reported to significantly stimulate hair shaft elongation, providing direct evidence of its hair-promoting activity at the organ level. In contrast, although FGF-10 (KGF-2) and FGF-2 (bFGF) have been associated with hair-related cellular responses, the corresponding human data are primarily derived from isolated follicle-associated cell cultures, animal models, or clinical delivery studies, rather than whole human hair follicle organ culture. Therefore, comparative efficacy among FGFs is discussed according to experimental model and strength of evidence, rather than as a definitive quantitative ranking within human hair follicle organ culture.

### 4.2. Animal Models: FGF-7 in Hair Growth

Animal studies have provided direct evidence for the functional role of FGF-7 in regulating the hair follicle cycle and promoting hair regeneration.

In C57BL/6 mouse models, exogenous administration of FGF-7 (either via topical application or injection) significantly shortens the telogen phase and accelerates the transition of hair follicles into the anagen phase, resulting in enhanced follicular activation and increased hair density. Early studies further demonstrated that the topical application of recombinant FGF-7 to mouse skin markedly stimulates follicular proliferation and keratinocyte activity [37].

Transgenic mouse models have further reinforced the indispensable role of FGF-7 in hair regeneration. Guo et al. showed that FGF-7 is crucial during skin development, as its overexpression accelerates hair follicle regeneration. In contrast, deletion or inhibition of FGF-7 leads to abnormal, sparse hair growth. These findings showed the essential function of FGF-7 in maintaining and promoting the proliferation of hair follicle epithelial cells [62]. Several plant-derived natural extracts have been found to indirectly upregulate FGF-7 expression and enhance hair growth. For instance, the BeauTop formulation, comprising multiple traditional Chinese herbal ingredients, significantly increased FGF-7 mRNA levels in the skin and enhanced follicle density in a rat model [65]. Similarly, recent studies have reported that the BFNB complex elevates EGF and FGF-7 expression in C57BL/6 mice, activating the PI3K-AKT-β-catenin signaling pathway and ultimately stimulating hair regeneration [66].

### 4.3. Human Studies: FGF-7 Expression and Preliminary Clinical Evidence

To date, no randomized controlled trials in humans have directly evaluated the effects of exogenous FGF-7 (e.g., recombinant palifermin) with quantitative outcomes or clinical endpoints as primary measures. However, one study reported that topical application of a 2% pea sprout extract to the scalps of 10 volunteers resulted in an approximately 56% increase in FGF-7 gene expression and a significant reduction in hair loss over 8 weeks. This study represents one of the few clinical investigations specifically targeting FGF-7 in the context of hair growth and hair loss [67]. Although topical or cosmetic interventions have been reported to increase FGF-7 mRNA expression in human scalp samples, the magnitude of upregulation required to induce a biologically meaningful anagen response in human follicles remains undefined. Such transcript-level changes should therefore be interpreted as hypothesis-generating biomarkers, rather than as organ-level functional endpoints (e.g., anagen induction or hair shaft elongation), because intrafollicular FGF-7 protein availability has not been directly quantified. In contrast, evidence from human hair follicle organ culture demonstrates that exogenous FGF-7 can promote hair shaft elongation, supporting its biological activity at the follicular level.

## 5. Therapeutic and Cosmetic Applications

### 5.1. Recombinant FGF-7

Clinical and Skin Repair Evidence

Recombinant FGF-7 (palifermin) has been clinically applied as an epithelial protective and regenerative agent to mitigate severe chemotherapy-induced oral mucositis. Numerous reviews and experimental studies have confirmed its epithelium-specific activity and its capacity to promote re-epithelialization [68,69]. In dermatology and wound healing, FGF-7 plays a key role in epidermal regeneration and scar inhibition, facilitating both re-epithelialization and granulation tissue formation. Recent approaches have further explored FGF-7 gene delivery and biomaterial-based FGF-7 release systems to accelerate the rate and quality of wound healing [68,70,71,72].

### 5.2. Biomaterials & Delivery Systems

#### 5.2.1. Technical Challenges of Topical Scalp Application

Direct topical delivery of FGF-7 to the scalp is limited by the barrier function of the stratum corneum and the inherent instability of protein therapeutics, posing challenges for both skin penetration and drug stability. Recent advances in microneedle technology and transdermal delivery systems have begun to address these limitations. Among available strategies, microneedle-assisted delivery and follicular-targeting particulate carriers appear particularly promising for achieving intrafollicular deposition by bypassing the stratum corneum barrier and exploiting the hair follicle as a drug reservoir. Approaches include microneedle patches designed for loading and controlled release of multiple FGFs, as well as electrostimulation-assisted microneedle systems, both of which significantly improve dermal penetration and bioavailability in the skin. These versatile delivery platforms can be readily adapted for the administration of FGF-7 [73].

#### 5.2.2. Nanoparticle and Hydrogel Carriers

Research has demonstrated that biomaterials and protein engineering approaches can maintain growth factor activity, extend in situ half-life, and enable sustained, localized release. Strategies targeting FGF-7 for wound repair, including hydrogels, microparticles, and gene delivery systems, have been demonstrated to enhance healing outcomes in animal and preclinical models. Although the majority of studies have utilized wound-healing models, the underlying principles of protein stabilization and controlled release are directly applicable to the scalp microenvironment [68,74].

#### 5.2.3. Exosomes and Plant-Derived Exosomes as Novel Carriers

Exosomes, owing to their capacity for intercellular delivery and membrane fusion, are emerging as a promising platform for targeted delivery to the scalp and hair follicles to promote regeneration. Clinical and preclinical studies on hair follicle regeneration and hair loss therapy are rapidly expanding, with most investigations using mesenchymal stem cell-derived exosomes. Early human studies have reported improvements in hair count and density; however, product quality and standardization remain variable [75,76,77]. Plant-derived exosome-like nanoparticles have also been proposed as a safe and scalable carrier, with growing evidence supporting their potential applications in skin and regenerative medicine. In the context of human hair, current studies are largely focused on proof-of-concept and feasibility [78].

## 6. Safety Considerations

### 6.1. Potential Risks—Proliferation and Tumor Safety

The primary concern with growth factor-based therapies is the potential to induce excessive epithelial cell proliferation and to initiate or promote tumor progression. Clinically, human recombinant FGF-7 (palifermin) has been evaluated in multiple trials and reviews and demonstrates an acceptable safety profile when used to prevent chemotherapy- or radiotherapy-induced oral mucositis. Adverse effects are generally mild and reversible, including rash, pruritus, and taste alterations, with no clear evidence of tumor-promoting activity in humans [79]. Moreover, in vitro and preclinical studies have not demonstrated that palifermin stimulates the growth of KGFR-expressing tumor cells or protects tumors from the cytotoxic effects of chemotherapy. Nevertheless, a theoretical risk of tumorigenesis remains, requiring rigorous clinical evaluation and long-term monitoring [80]. Therefore, extrapolation of systemic palifermin safety data to chronic topical scalp exposure should be approached cautiously, as topical administration may result in distinct local exposure kinetics. Although systemic palifermin experience is generally reassuring within its approved clinical contexts, evidence implicating FGF/FGFR signaling in subsets of cutaneous malignancies suggests that long-term topical use should incorporate careful patient selection, conservative dosing strategies, and post-marketing safety surveillance. Currently, there is no direct evidence linking FGF-7 to skin tumor formation; however, FGFR signaling may contribute to tumor progression in certain skin cancers, such as cutaneous squamous cell carcinoma. Thus, therapeutic interventions targeting FGF/FGFR pathways should be carefully optimized with respect to dosage, exposure duration, and clinical indications [81].

### 6.2. Route of Administration—Topical vs. Systemic

Topical formulations containing growth factors, whether cosmetic or dermocosmetic, generally result in minimal systemic exposure. Recent systematic reviews of topical growth factors used for facial rejuvenation have not identified major short- to medium-term safety concerns. However, the available evidence is limited mainly to small, non-randomized studies, and data on long-term use and potential carcinogenic risk remain insufficient. Consequently, while topical administration appears relatively safe, ongoing monitoring is warranted [82]. In contrast, systemic administration (e.g., intravenous palifermin) utilizes pharmaceutical-grade preparations. Reported adverse events are primarily rash, pruritus, erythema, oral and tongue disorders, and taste alterations; these effects are generally mild to moderate in severity and transient [69].

### 6.3. Future Perspectives

FGF-7 has emerged as a promising candidate for hair follicle regeneration; however, several challenges must be addressed before clinical translation. Future research may focus on the development of advanced delivery systems, such as microneedles, hydrogels, and nanocarriers, to achieve controlled, targeted release within the hair follicle microenvironment while minimizing systemic exposure [83,84,85]. Rational combinations of FGF-7 with existing therapies may further enhance efficacy. For example, minoxidil can upregulate growth factor signaling [86], platelet-rich plasma directly enhances FGF-7 expression and promotes angiogenesis [87], and low-level laser therapy modulates follicular stem cell activity and sustains the anagen phase through photobiomodulation [88]. Such combinational approaches have the potential to amplify therapeutic outcomes via complementary mechanisms.

Pharmacokinetic improvements in FGF-7 through fusion with antibody fragment crystallizable region (Fc-fusion) [89] or PEGylation [90] represent a promising approach to extend its half-life and reduce dosing frequency. Gene- and mRNA-based approaches also hold considerable potential by enabling localized and sustained FGF-7 expression within the follicular region; notably, recent mRNA therapeutics for skin regeneration have demonstrated encouraging efficacy [91]. Emerging strategies such as FGF-7 mimetics (small molecules or peptides) and gene therapy offer additional avenues for intervention. Ligand engineering and carrier modification, such as site-specific mutation or PEGylation of recombinant human FGF-7, can further enhance molecular stability and receptor selectivity while maintaining bioactivity [92,93]. Furthermore, nucleic acid aptamers and peptides targeting FGFR2b have been developed to precisely modulate downstream signaling [94]. In parallel, gene delivery systems employing adenoviral, adeno-associated viral, or lipid nanoparticle vectors enable localized FGF-7 expression within hair follicles, offering a novel platform for regeneration, although their long-term safety and immunogenicity remain to be further evaluated and fully characterized [95,96,97]. EX104 is an engineered exosome designed to simultaneously deliver WNT10B, VEGFA, and FGF-7 to counteract DHT-induced hair follicle miniaturization. In a murine model of androgenetic alopecia, topical EX104 application significantly promoted hair growth, showing comparable efficacy to Minoxidil and superior effects on angiogenesis and follicular proliferation [98]. Encapsulation of FGF-7 within exosomes improves its follicular delivery and strengthens its hair-promoting activity. In another study, umbilical cord blood-derived mesenchymal stem cells (UCB-MSCs) were transfected with a specific miR-31-5p inhibitor, and the exosomes derived from these cells were collected. These modified exosomes upregulated FGF-7 expression and significantly enhanced the proliferation, migration, and differentiation capacities of UCB-MSCs [99]. By utilizing exosomes as biological delivery vehicles, either to efficiently transport FGF-7 into the hair follicle microenvironment or to encapsulate a miR-31-5p inhibitor that alleviates its negative regulation on FGF-7 expression, marked enhancements in hair follicle cell proliferation, migration, and differentiation can be achieved, accompanied by improved hair regeneration. This exosome-mediated regulatory strategy represents a promising and highly translatable therapeutic approach for alopecia, potentially offering an innovative treatment modality for androgenetic alopecia and other hair-loss disorders.

On the research front, hair follicle organoids and stem cell-based models offer powerful platforms for investigating the roles of FGF-7 in follicle induction and cycling, thereby facilitating its clinical translation in follicular tissue engineering [100]. The integration of single-cell transcriptomic and proteomic approaches will further delineate FGF-7–mediated signaling networks and identify novel therapeutic targets [101,102]. Clinical translation should also consider the alopecia subtype. AGA reflects chronic follicular miniaturization with altered progenitor dynamics [12], whereas CIA represents acute cytotoxic injury to matrix keratinocytes. Direct comparative data evaluating differential responsiveness to FGF-7 between these conditions are currently lacking, underscoring the need for etiology-stratified clinical trials. Evaluation of FGF-7-based interventions should incorporate multi-level biomarkers, including clinical endpoints (hair density, shaft diameter, shedding counts), follicle-level measures (anagen-to-telogen ratio, hair shaft elongation in organ culture), and molecular indicators of epithelial activation and follicular regeneration (e.g., Ki-67, K15/K19, Wnt/β-catenin markers, and VEGF-associated vascular indices). In summary, these emerging strategies represent the multifaceted potential of FGF-7 as a cornerstone in the development of next-generation hair regeneration therapies.

## 7. Conclusions

FGF-7 plays a pivotal role in hair follicle physiology and regeneration. As a key mesenchymal–epithelial signaling molecule, FGF-7 promotes keratinocyte proliferation and supports the transition of hair follicles into the anagen phase [62,63,64]. Accumulating evidence from in vitro and animal studies demonstrates its capacity to stimulate follicular growth and protect follicular cells from apoptosis [22,101]. However, clinical evidence supporting the efficacy of FGF-7–based interventions in alopecia remains limited, underscoring the need for rigorously designed randomized controlled trials.

Despite its promising biological activity, several critical knowledge gaps must be addressed before FGF-7–based therapies can be translated into routine clinical practice. These include the definition of optimal dosing regimens and duration of exposure required to achieve sustained follicular responses, the development of efficient follicle-targeted delivery strategies that maximize intrafollicular bioavailability while minimizing off-target effects, and the establishment of reliable and clinically meaningful biomarkers spanning molecular, follicular, and clinical endpoints to monitor therapeutic response and guide treatment optimization.

In addition, although topical FGF-7 has not been associated with major safety concerns to date, comprehensive evaluation of its long-term and systemic safety remains imperative. Clinical experience from oncology, where recombinant FGF-7 (palifermin) has been used therapeutically, highlights the importance of precise dose regulation and continuous safety surveillance [80,81]. Looking forward, FGF-7 holds considerable promise as a translational bridge between regenerative medicine and the cosmetic field, with potential applications including incorporation into biomaterial-based carriers, strategies to extend its biological half-life, and rational combination approaches with existing hair growth therapies [84,86,89,90]. Collectively, addressing these challenges will be pivotal for the rational development and safe clinical implementation of next-generation FGF-7–based interventions for hair regeneration.

## Figures and Tables

**Table 1:** Growth Factors and Their Roles in Hair Follicle Cycle.

Growth Factor	Secretory Cell	Target Cell	Representative Mechanisms	Net Effect on the Hair Cycle	References
FGF-7(KGF)	Dermal fibroblasts (stimulated by IL-1)	Hair follicle epithelial cells/keratinocytes (FGFR2b)	Stimulates proliferation and repair; activates ERK, AKT; IL-1 → KGF → Bipolar secretion of keratinocytes	Promotes/ maintain anagen	[30,37,40]
FGF-2	DPC, Mesenchymal	DPC, Keratinocyte	Promotes proliferation, survival, and microenvironment support; synergizes with PDGF to maintain inductive signals	Promotes growth (supportive)	[41]
FGF-5	Outer root sheath (late anagen ascending phase)	Hair follicular epithelial/Mesenchymal	Terminates signals and drives anagen → catagen transition	promote catagen (inhibit anagen)	[42,43]
FGF-9	γδ T cells (after injury)	Mesenchymal/epithelial	Promotes WIHN; enhances Wnt activity	Promotes neogenesis/regeneration	[44]
FGF-10	Mesenchymal/ Fibroblast	Epithelial (FGFR2b)	Supports epithelial development and hair follicle morphogenesis	Promotes development/regeneration	[45]
FGF-18	Hair follicle epithelium (high expression in telogen)	Epithelial/micro-environment	Maintains telogen and inhibits premature anagen	Maintains telogen/inhibits anagen	[28]
FGF-22	Inner root sheath (IRS)	Local epithelial structure	Structure-related expression	Unclear (structure-related)	[46]
IGF-1	DPC, Mesenchymal	Epithelial and DPC	Anti-apoptotic (↑Bcl-2/↓Bax), promotes proliferation; upregulates VEGF	Extends anagen/promotes growth	[16,47]
VEGF	Mesenchymal cell, DPC, endothelial	Microvascular endothelium/perifollicular	Angiogenesis and increased perfusion → promotes hair growth response	Promotes growth (vascular axis)	[17]
EGF	Epithelial, Mesenchymal	Epithelial/outer root sheath	Dose/timing dependent: promotes repair or induces catagen	Bidirectional regulation	[48,49]
PDGF	Platelet, DPC/Mesenchymal	DPC, Mesenchymal, Epithelial	Supports DPC proliferation and induction; synergizes with FGF-2	Promotes growth (supportive)	[41]
TGF-β2	Epithelial/Mesenchymal	Epithelial	Promotes apoptosis and catagen induction	Promotes catagen (growth inhibition)	[50]
IL-1 (→FGF-7 amplification)	Keratinocyte	Fibroblast (induces KGF)	IL-1 upregulates KGF/FGF-7 → epithelial proliferation	Indirectly promotes anagen/repair	[40]

## Data Availability

No new data were created or analyzed in this study. Data sharing is not applicable to this article.

## Abbreviations

AGA	Androgenetic alopecia
CIA	Chemotherapy-induced alopecia
DPC	Dermal papilla cell
EGF	Epidermal growth factor
FGF-7	Fibroblast Growth Factor 7
FGFR2b	Fibroblast growth factor receptor 2b
IGF-1	Insulin-like growth factor 1
HFSC	Hair follicle stem cell
PEG	Polyethylene glycol
PDGF	Platelet-Derived Growth Factor
TGF-β	Transforming Growth Factor-β
VEGF	Vascular Endothelial Growth Factor

## References

[1] Devjani S., Ezemma O., Kelley K.J., Stratton E., Senna M. (2023). Androgenetic Alopecia: Therapy Update. Drugs.

[2] Asghar F., Shamim N., Farooque U., Sheikh H., Aqeel R. (2020). Telogen Effluvium: A Review of the Literature. Cureus.

[3] Trueb R.M. (2009). Chemotherapy-induced alopecia. Semin. Cutan. Med. Surg..

[4] Huang C.H., Fu Y., Chi C.C. (2021). Health-Related Quality of Life, Depression, and Self-esteem in Patients With Androgenetic Alopecia: A Systematic Review and Meta-analysis. JAMA Dermatol..

[5] Wikramanayake T.C., Haberland N.I., Akhundlu A., Laboy Nieves A., Miteva M. (2023). Prevention and Treatment of Chemotherapy-Induced Alopecia: What Is Available and What Is Coming?. Curr. Oncol..

[6] Saraswat N., Chopra A., Sood A., Kamboj P., Kumar S. (2019). A Descriptive Study to Analyze Chemotherapy-Induced Hair Loss and its Psychosocial Impact in Adults: Our Experience from a Tertiary Care Hospital. Indian Dermatol. Online J..

[7] Hesketh P.J., Batchelor D., Golant M., Lyman G.H., Rhodes N., Yardley D. (2004). Chemotherapy-induced alopecia: Psychosocial impact and therapeutic approaches. Support. Care Cancer.

[8] Ben Kridis W., Boudawara O., Khanfir A. (2024). Chemotherapy induced alopecia in breast cancer patients: A monocentric prospective study. Breast Dis..

[9] Rossi A., Cantisani C., Melis L., Iorio A., Scali E., Calvieri S. (2012). Minoxidil use in dermatology, side effects and recent patents. Recent Pat. Inflamm. Allergy Drug Discov..

[10] Gupta A.K., Venkataraman M., Talukder M., Bamimore M.A. (2022). Finasteride for hair loss: A review. J. Dermatolog. Treat..

[11] Cuevas-Diaz Duran R., Martinez-Ledesma E., Garcia-Garcia M., Gauzin D.B., Sarro-Ramírez A., Gonzalez-Carrillo C., Rodríguez-Sardin D., Fuentes A., Cardenas-Lopez A. (2024). The Biology and Genomics of Human Hair Follicles: A Focus on Androgenetic Alopecia. Int. J. Mol. Sci..

[12] Garza L.A., Yang C.C., Zhao T., Blatt H.B., Lee M., He H., Stanton D.C., Carrasco L., Spiegel J.H., Tobias J.W. (2011). Bald scalp in men with androgenetic alopecia retains hair follicle stem cells but lacks CD200-rich and CD34-positive hair follicle progenitor cells. J. Clin. Investig..

[13] Malkud S. (2015). Telogen Effluvium: A Review. J. Clin. Diagn. Res..

[14] Paus R., Haslam I.S., Sharov A.A., Botchkarev V.A. (2013). Pathobiology of chemotherapy-induced hair loss. Lancet Oncol..

[15] Freites-Martinez A., Shapiro J., van den Hurk C., Goldfarb S., Jimenez J.J., Rossi A.M., Paus R., Lacouture M.E. (2019). Hair disorders in cancer survivors. J. Am. Acad. Dermatol..

[16] Hsieh W.J., Qiu W.Y., Percec I., Chang T.M. (2025). Insulin-like Growth Factor 1 (IGF-1) in Hair Regeneration: Mechanistic Pathways and Therapeutic Potential. Curr. Issues Mol. Biol..

[17] Yano K., Brown L.F., Detmar M. (2001). Control of hair growth and follicle size by VEGF-mediated angiogenesis. J. Clin. Investig..

[18] Mak K.K., Chan S.Y. (2003). Epidermal growth factor as a biologic switch in hair growth cycle. J. Biol. Chem..

[19] Hebert J.M., Rosenquist T., Gotz J., Martin G.R. (1994). FGF5 as a regulator of the hair growth cycle: Evidence from targeted and spontaneous mutations. Cell.

[20] Rosenquist T.A., Martin G.R. (1996). Fibroblast growth factor signalling in the hair growth cycle: Expression of the fibroblast growth factor receptor and ligand genes in the murine hair follicle. Dev. Dyn..

[21] Grose R., Fantl V., Werner S., Chioni A.M., Jarosz M., Rudling R., Cross B., Hart I.R., Dickson C. (2007). The role of fibroblast growth factor receptor 2b in skin homeostasis and cancer development. EMBO J..

[22] Braun S., Krampert M., Bodo E., Kumin A., Born-Berclaz C., Paus R., Werner S. (2006). Keratinocyte growth factor protects epidermis and hair follicles from cell death induced by UV irradiation, chemotherapeutic or cytotoxic agents. J. Cell Sci..

[23] Zinkle A., Mohammadi M. (2019). Structural Biology of the FGF7 Subfamily. Front. Genet..

[24] Edirisinghe O., Ternier G., Alraawi Z., Suresh Kumar T.K. (2024). Decoding FGF/FGFR Signaling: Insights into Biological Functions and Disease Relevance. Biomolecules.

[25] Wang N., Zhang W., Zhong Z., Zhou X., Shi X., Wang X. (2025). FGF7 secreted from dermal papillae cell regulates the proliferation and differentiation of hair follicle stem cell. J. Integr. Agric..

[26] Kinoshita-Ise M., Tsukashima A., Kinoshita T., Yamazaki Y., Ohyama M. (2020). Altered FGF expression profile in human scalp-derived fibroblasts upon WNT activation: Implication of their role to provide folliculogenetic microenvironment. Inflamm. Regen..

[27] Rosato B., Ranieri D., Nanni M., Torrisi M.R., Belleudi F. (2018). Role of FGFR2b expression and signaling in keratinocyte differentiation: Sequential involvement of PKCdelta and PKCalpha. Cell Death Dis..

[28] Kawano M., Komi-Kuramochi A., Asada M., Suzuki M., Oki J., Jiang J., Imamura T. (2005). Comprehensive analysis of FGF and FGFR expression in skin: FGF18 is highly expressed in hair follicles and capable of inducing anagen from telogen stage hair follicles. J. Investig. Dermatol..

[29] Kurban G., Ishiwata T., Kudo M., Yokoyama M., Sugisaki Y., Naito Z. (2004). Expression of keratinocyte growth factor receptor (KGFR/FGFR2 IIIb) in human uterine cervical cancer. Oncol. Rep..

[30] Epstein R.J., Tian L.J., Gu Y.F. (2021). 2b or Not 2b: How Opposing FGF Receptor Splice Variants Are Blocking Progress in Precision Oncology. J. Oncol..

[31] Niehues H., Rikken G., van Vlijmen-Willems I., Rodijk-Olthuis D., van Erp P.E.J., Zeeuwen P., Schalkwijk J., van den Bogaard E.H. (2022). Identification of Keratinocyte Mitogens: Implications for Hyperproliferation in Psoriasis and Atopic Dermatitis. JID Innov..

[32] Bao S., Wang Y., Sweeney P., Chaudhuri A., Doseff A.I., Marsh C.B., Knoell D.L. (2005). Keratinocyte growth factor induces Akt kinase activity and inhibits Fas-mediated apoptosis in A549 lung epithelial cells. Am. J. Physiol. Lung Cell Mol. Physiol..

[33] Niu J., Chang Z., Peng B., Xia Q., Lu W., Huang P., Tsao M.S., Chiao P.J. (2007). Keratinocyte growth factor/fibroblast growth factor-7-regulated cell migration and invasion through activation of NF-kappaB transcription factors. J. Biol. Chem..

[34] Waters C.M., Savla U., Panos R.J. (1997). KGF prevents hydrogen peroxide-induced increases in airway epithelial cell permeability. Am. J. Physiol..

[35] Philpott M.P., Sanders D., Westgate G.E., Kealey T. (1994). Human hair growth in vitro: A model for the study of hair follicle biology. J. Dermatol. Sci..

[36] Lin X., Zhu L., He J. (2022). Morphogenesis, Growth Cycle and Molecular Regulation of Hair Follicles. Front. Cell Dev. Biol..

[37] Danilenko D.M., Ring B.D., Yanagihara D., Benson W., Wiemann B., Starnes C.O., Pierce G.F. (1995). Keratinocyte growth factor is an important endogenous mediator of hair follicle growth, development, and differentiation. Normalization of the nu/nu follicular differentiation defect and amelioration of chemotherapy-induced alopecia. Am. J. Pathol..

[38] Quist S.R., Quist J. (2021). Keep quiet-how stress regulates hair follicle stem cells. Signal Transduct. Target. Ther..

[39] Woo W.M., Oro A.E. (2011). SnapShot: Hair follicle stem cells. Cell.

[40] Brauchle M., Angermeyer K., Hubner G., Werner S. (1994). Large induction of keratinocyte growth factor expression by serum growth factors and pro-inflammatory cytokines in cultured fibroblasts. Oncogene.

[41] Kiso M., Hamazaki T.S., Itoh M., Kikuchi S., Nakagawa H., Okochi H. (2015). Synergistic effect of PDGF and FGF2 for cell proliferation and hair inductive activity in murine vibrissal dermal papilla in vitro. J. Dermatol. Sci..

[42] Ota Y., Saitoh Y., Suzuki S., Ozawa K., Kawano M., Imamura T. (2002). Fibroblast growth factor 5 inhibits hair growth by blocking dermal papilla cell activation. Biochem. Biophys. Res. Commun..

[43] Higgins C.A., Petukhova L., Harel S., Ho Y.Y., Drill E., Shapiro L., Wajid M., Christiano A.M. (2014). FGF5 is a crucial regulator of hair length in humans. Proc. Natl. Acad. Sci. USA.

[44] Gay D., Kwon O., Zhang Z., Spata M., Plikus M.V., Holler P.D., Ito M., Yang Z., Treffeisen E., Kim C.D. (2013). Fgf9 from dermal gammadelta T cells induces hair follicle neogenesis after wounding. Nat. Med..

[45] Petiot A., Conti F.J., Grose R., Revest J.M., Hodivala-Dilke K.M., Dickson C. (2003). A crucial role for Fgfr2-IIIb signalling in epidermal development and hair follicle patterning. Development.

[46] Nakatake Y., Hoshikawa M., Asaki T., Kassai Y., Itoh N. (2001). Identification of a novel fibroblast growth factor, FGF-22, preferentially expressed in the inner root sheath of the hair follicle. Biochim. Biophys. Acta.

[47] Ahn S.Y., Pi L.Q., Hwang S.T., Lee W.S. (2012). Effect of IGF-I on Hair Growth Is Related to the Anti-Apoptotic Effect of IGF-I and Up-Regulation of PDGF-A and PDGF-B. Ann. Dermatol..

[48] Philpott M.P., Kealey T. (1994). Effects of EGF on the morphology and patterns of DNA synthesis in isolated human hair follicles. J. Investig. Dermatol..

[49] Paik S.H., Yoon J.S., Ryu H.H., Lee J.Y., Shin C.Y., Min K.H., Jo S.J., Kim K.H., Kwon O. (2013). Pretreatment of epidermal growth factor promotes primary hair recovery via the dystrophic anagen pathway after chemotherapy-induced alopecia. Exp. Dermatol..

[50] Soma T., Tsuji Y., Hibino T. (2002). Involvement of transforming growth factor-beta2 in catagen induction during the human hair cycle. J. Investig. Dermatol..

[51] Maas-Szabowski N., Shimotoyodome A., Fusenig N.E. (1999). Keratinocyte growth regulation in fibroblast cocultures via a double paracrine mechanism. J. Cell Sci..

[52] Addis D.R., Pan L., Musicaro R., Schacter D.L. (2016). Divergent thinking and constructing episodic simulations. Memory.

[53] Stadelmann N., Horch R.E., Schmid R., Ostendorf D., Peddi A., Promny T., Boos A.M., Kengelbach-Weigand A. (2025). Growth factors IGF-1 and KGF and adipose-derived stem cells promote migration and viability of primary human keratinocytes in an in vitro wound model. Front. Med..

[54] Li J., Yang Z., Li Z., Gu L., Wang Y., Sung C. (2014). Exogenous IGF-1 promotes hair growth by stimulating cell proliferation and down regulating TGF-beta1 in C57BL/6 mice in vivo. Growth Horm. IGF Res..

[55] Li W., Man X.Y., Li C.M., Chen J.Q., Zhou J., Cai S.Q., Lu Z.F., Zheng M. (2012). VEGF induces proliferation of human hair follicle dermal papilla cells through VEGFR-2-mediated activation of ERK. Exp. Cell Res..

[56] Mecklenburg L., Tobin D.J., Müller-Röver S., Handjiski B., Wendt G., Peters E.M., Pohl S., Moll I., Paus R. (2000). Active hair growth (anagen) is associated with angiogenesis. J. Investig. Dermatol..

[57] Richardson G.D., Bazzi H., Fantauzzo K.A., Waters J.M., Crawford H., Hynd P., Christiano A.M., Jahoda C.A. (2009). KGF and EGF signalling block hair follicle induction and promote interfollicular epidermal fate in developing mouse skin. Development.

[58] Philpott M.P., Sanders D.A., Kealey T. (1994). Effects of insulin and insulin-like growth factors on cultured human hair follicles: IGF-I at physiologic concentrations is an important regulator of hair follicle growth in vitro. J. Investig. Dermatol..

[59] Tobin D.J. (2011). Ex vivo organ culture of human hair follicles: A model epithelial-neuroectodermal-mesenchymal interaction system. Methods Mol. Biol..

[60] Tsuboi R., Sato C., Kurita Y., Ron D., Rubin J.S., Ogawa H. (1993). Keratinocyte growth factor (FGF-7) stimulates migration and plasminogen activator activity of normal human keratinocytes. J. Investig. Dermatol..

[61] Maretzky T., Evers A., Zhou W., Swendeman S.L., Wong P.M., Rafii S., Reiss K., Blobel C.P. (2011). Migration of growth factor-stimulated epithelial and endothelial cells depends on EGFR transactivation by ADAM17. Nat. Commun..

[62] Guo L., Degenstein L., Fuchs E. (1996). Keratinocyte growth factor is required for hair development but not for wound healing. Genes Dev..

[63] McCormack B. (2009). Editorial. Int. J. Older People Nurs..

[64] Iino M., Ehama R., Nakazawa Y., Iwabuchi T., Ogo M., Tajima M., Arase S. (2007). Adenosine stimulates fibroblast growth factor-7 gene expression via adenosine A2b receptor signaling in dermal papilla cells. J. Investig. Dermatol..

[65] Lee C.Y., Yang C.Y., Lin C.C., Yu M.C., Sheu S.J., Kuan Y.H. (2018). Hair growth is promoted by BeauTop via expression of EGF and FGF-7. Mol. Med. Rep..

[66] Perez-Mora S., Ocampo-Lopez J., Gomez-Garcia M.D.C., Perez-Ishiwara D.G. (2023). BFNB Enhances Hair Growth in C57BL/6 Mice through the Induction of EGF and FGF7 Factors and the PI3K-AKT-beta-Catenin Pathway. Int. J. Mol. Sci..

[67] Grothe T., Wandrey F., Schuerch C. (2020). Short communication: Clinical evaluation of pea sprout extract in the treatment of hair loss. Phytother. Res..

[68] Bartolo I., Reis R.L., Marques A.P., Cerqueira M.T. (2022). Keratinocyte Growth Factor-Based Strategies for Wound Re-Epithelialization. Tissue Eng. Part B Rev..

[69] Spielberger R., Stiff P., Bensinger W., Gentile T., Weisdorf D., Kewalramani T., Shea T., Yanovich S., Hansen K., Noga S. (2004). Palifermin for oral mucositis after intensive therapy for hematologic cancers. N. Engl. J. Med..

[70] Beer H.D., Gassmann M.G., Munz B., Steiling H., Engelhardt F., Bleuel K., Werner S. (2000). Expression and function of keratinocyte growth factor and activin in skin morphogenesis and cutaneous wound repair. J. Investig. Dermatol. Symp. Proc..

[71] Finch P.W., Mark Cross L.J., McAuley D.F., Farrell C.L. (2013). Palifermin for the protection and regeneration of epithelial tissues following injury: New findings in basic research and pre-clinical models. J. Cell Mol. Med..

[72] Barrientos S., Brem H., Stojadinovic O., Tomic-Canic M. (2014). Clinical application of growth factors and cytokines in wound healing. Wound Repair Regen..

[73] Yang G., Hu S., Jiang H., Cheng K. (2023). Peelable Microneedle Patches Deliver Fibroblast Growth Factors to Repair Skin Photoaging Damage. Nanotheranostics.

[74] Tallapaneni V., Mude L., Pamu D., Palanimuthu V.R., Magham S.V., Karri V., Parvathaneni M. (2022). Growth Factor Loaded Thermo-Responsive Injectable Hydrogel for Enhancing Diabetic Wound Healing. Gels.

[75] Gupta A.K., Wang T., Rapaport J.A. (2023). Systematic review of exosome treatment in hair restoration: Preliminary evidence, safety, and future directions. J. Cosmet. Dermatol..

[76] Cheng M., Ma C., Chen H.D., Wu Y., Xu X.G. (2024). The Roles of Exosomes in Regulating Hair Follicle Growth. Clin. Cosmet. Investig. Dermatol..

[77] Queen D., Avram M.R. (2025). Exosomes for Treating Hair Loss: A Review of Clinical Studies. Dermatol. Surg..

[78] Zhu Y., Zhao J., Ding H., Qiu M., Xue L., Ge D., Wen G., Ren H., Li P., Wang J. (2024). Applications of plant-derived extracellular vesicles in medicine. MedComm.

[79] Vadhan-Raj S., Goldberg J.D., Perales M.A., Berger D.P., van den Brink M.R. (2013). Clinical applications of palifermin: Amelioration of oral mucositis and other potential indications. J. Cell Mol. Med..

[80] Hille A., Gruger S., Christiansen H., Wolff H.A., Volkmer B., Lehmann J., Dorr W., Rave-Frank M. (2010). Effect of tumour-cell-derived or recombinant keratinocyte growth factor (KGF) on proliferation and radioresponse of human epithelial tumour cells (HNSCC) and normal keratinocytes in vitro. Radiat. Environ. Biophys..

[81] Khandelwal A.R., Kent B., Hillary S., Alam M.M., Ma X., Gu X., DiGiovanni J., Nathan C.O. (2019). Fibroblast growth factor receptor promotes progression of cutaneous squamous cell carcinoma. Mol. Carcinog..

[82] Quinlan D.J., Ghanem A.M., Hassan H. (2023). Topical growth factor preparations for facial skin rejuvenation: A systematic review. J. Cosmet. Dermatol..

[83] Ding Y.W., Li Y., Zhang Z.W., Dao J.W., Wei D.X. (2024). Hydrogel forming microneedles loaded with VEGF and Ritlecitinib/polyhydroxyalkanoates nanoparticles for mini-invasive androgenetic alopecia treatment. Bioact. Mater..

[84] Lei Y., Jiang W., Peng C., Wu D., Wu J., Xu Y., Yan H., Xia X. (2024). Advances in polymeric nano-delivery systems targeting hair follicles for the treatment of acne. Drug Deliv..

[85] Cao J., Wu B., Yuan P., Liu Y., Hu C. (2024). Advances in Research of Hydrogel Microneedle-Based Delivery Systems for Disease Treatment. Pharmaceutics.

[86] Choi N., Shin S., Song S.U., Sung J.H. (2018). Minoxidil Promotes Hair Growth through Stimulation of Growth Factor Release from Adipose-Derived Stem Cells. Int. J. Mol. Sci..

[87] Gentile P., Scioli M.G., Bielli A., De Angelis B., De Sio C., De Fazio D., Ceccarelli G., Trivisonno A., Orlandi A., Cervelli V. (2019). Platelet-Rich Plasma and Micrografts Enriched with Autologous Human Follicle Mesenchymal Stem Cells Improve Hair Re-Growth in Androgenetic Alopecia. Biomolecular Pathway Analysis and Clinical Evaluation. Biomedicines.

[88] Pillai J.K., Mysore V. (2021). Role of Low-Level Light Therapy (LLLT) in Androgenetic Alopecia. J. Cutan. Aesthetic Surg..

[89] Czajkowsky D.M., Hu J., Shao Z., Pleass R.J. (2012). Fc-fusion proteins: New developments and future perspectives. EMBO Mol. Med..

[90] Gao Y., Joshi M., Zhao Z., Mitragotri S. (2024). PEGylated therapeutics in the clinic. Bioeng. Transl. Med..

[91] Denzinger M., Link A., Kurz J., Krauss S., Thoma R., Schlensak C., Wendel H.P., Krajewski S. (2018). Keratinocyte Growth Factor Modified Messenger RNA Accelerating Cell Proliferation and Migration of Keratinocytes. Nucleic Acid. Ther..

[92] Poorebrahim M., Sadeghi S., Ghorbani R., Asghari M., Abazari M.F., Kalhor H., Rahimi H. (2017). In silico enhancement of the stability and activity of keratinocyte growth factor. J. Theor. Biol..

[93] Huang Z., Zhu G., Sun C., Zhang J., Zhang Y., Zhang Y., Ye C., Wang X., Ilghari D., Li X. (2012). A novel solid-phase site-specific PEGylation enhances the in vitro and in vivo biostabilty of recombinant human keratinocyte growth factor 1. PLoS ONE.

[94] Biggs M.A., Das A., Goncalves B.G., Murray M.E., Frantzeskos S.A., Hunt H.L., Phan C.A.N., Banerjee I.A. (2024). Developing New Peptides and Peptide-Drug Conjugates for Targeting the FGFR2 Receptor-Expressing Tumor Cells and 3D Spheroids. Biomimetics.

[95] Ohyama M., Vogel J.C. (2003). Gene delivery to the hair follicle. J. Investig. Dermatol. Symp. Proc..

[96] Lin C., Greenblatt M.B., Gao G., Shim J.H. (2024). Development of AAV-Mediated Gene Therapy Approaches to Treat Skeletal Diseases. Hum. Gene Ther..

[97] Guri-Lamce I., AlRokh Y., Kim Y., Maeshima R., Graham C., Hart S.L., McGrath J.A., Jackow-Malinowska J. (2024). Topical gene editing therapeutics using lipid nanoparticles: ‘gene creams’ for genetic skin diseases?. Br. J. Dermatol..

[98] Liu Y., Liu Y., Zhao J., Deng T., Ben Y., Lu R., Zhou X., Yan R., Chen X., Zhang J.V. (2025). Subcutaneous injection of genetically engineered exosomes for androgenic alopecia treatment. Front. Bioeng. Biotechnol..

[99] Liu S., Zhang W., Deng C., Wang H. (2025). Effects of miR-210-3p/SDF2 and miR-31-5p/FGF7 from hypoxic endometrial exosomes on UCB-MSC proliferation, migration, and differentiation. Stem Cell Res. Ther..

[100] Chu X., Zhou Z., Qian X., Shen H., Cheng H., Zhang J. (2025). Functional regeneration strategies of hair follicles: Advances and challenges. Stem Cell Res. Ther..

[101] Wang J., Wang L., Gao S., Li X. (2025). Recent Advances in the Role of Fibroblast Growth Factors in Hair Follicle Growth. Biomolecules.

[102] Li Y., Dong T., Wan S., Xiong R., Jin S., Dai Y., Guan C. (2024). Application of multi-omics techniques to androgenetic alopecia: Current status and perspectives. Comput. Struct. Biotechnol. J..

